# ANGPTL3 deficiency impairs lipoprotein production and produces adaptive changes in hepatic lipid metabolism

**DOI:** 10.1016/j.jlr.2024.100500

**Published:** 2024-01-14

**Authors:** Kendall H. Burks, Yan Xie, Michael Gildea, In-Hyuk Jung, Sandip Mukherjee, Paul Lee, Upasana Pudupakkam, Ryan Wagoner, Ved Patel, Katherine Santana, Arturo Alisio, Ira J. Goldberg, Brian N. Finck, Edward A. Fisher, Nicholas O. Davidson, Nathan O. Stitziel

**Affiliations:** 1 Division of Cardiology, Department of Medicine, Center for Cardiovascular Research, Washington University School of Medicine, Saint Louis, MO, USA; 2 Division of Gastroenterology, Department of Medicine, Washington University School of Medicine, Saint Louis, MO, USA; 3 Division of Cardiology, Department of Medicine, New York University Grossman School of Medicine, New York, NY, USA; 4 Division of Nutritional Science and Obesity Medicine, Department of Medicine, Center for Human Nutrition, Washington University School of Medicine, Saint Louis, MO, USA; 5 Division of Endocrinology, Diabetes and Metabolism, Department of Medicine, New York University Grossman School of Medicine, New York, NY, USA; 6 Department of Genetics, Washington University School of Medicine, Saint Louis, MO, USA

**Keywords:** apolipoproteins, dyslipidemias, LDL/metabolism, lipase/endothelial, PPARs, triglycerides, VLDL

## Abstract

Angiopoietin-like protein 3 (ANGPTL3) is a hepatically secreted protein and therapeutic target for reducing plasma triglyceride-rich lipoproteins and low-density lipoprotein (LDL) cholesterol. Although ANGPTL3 modulates the metabolism of circulating lipoproteins, its role in triglyceride-rich lipoprotein assembly and secretion remains unknown. CRISPR-associated protein 9 (CRISPR/Cas9) was used to target *ANGPTL3* in HepG2 cells (*ANGPTL3*^*−/−*^) whereupon we observed ∼50% reduction of apolipoprotein B100 (ApoB100) secretion, accompanied by an increase in ApoB100 early presecretory degradation via a predominantly lysosomal mechanism. Despite defective particle secretion in *ANGPTL3*^*−/−*^ cells, targeted lipidomic analysis did not reveal neutral lipid accumulation in *ANGPTL3*^*−/−*^ cells; rather *ANGPTL3*^*−/−*^ cells demonstrated decreased secretion of newly synthesized triglycerides and increased fatty acid oxidation. Furthermore, RNA sequencing demonstrated significantly altered expression of key lipid metabolism genes, including targets of peroxisome proliferator-activated receptor α, consistent with decreased lipid anabolism and increased lipid catabolism. In contrast, CRISPR/Cas9 LDL receptor (LDLR) deletion in *ANGPTL3*^*−/−*^ cells did not result in a secretion defect at baseline, but proteasomal inhibition strongly induced compensatory late presecretory degradation of ApoB100 and impaired its secretion. Additionally, these *ANGPTL3*^*−/−*^*;LDLR*^*−/−*^ cells rescued the deficient LDL clearance of *LDLR*^*−/−*^ cells. In summary, ANGPTL3 deficiency in the presence of functional LDLR leads to the production of fewer lipoprotein particles due to early presecretory defects in particle assembly that are associated with adaptive changes in intrahepatic lipid metabolism. In contrast, when LDLR is absent, ANGPTL3 deficiency is associated with late presecretory regulation of ApoB100 degradation without impaired secretion. Our findings therefore suggest an unanticipated intrahepatic role for ANGPTL3, whose function varies with LDLR status.

Angiopoietin-like protein 3 (ANGPTL3) is a glycoprotein secreted almost exclusively by hepatocytes that plays important roles in lipoprotein metabolism. Circulating ANGPTL3 inhibits both lipoprotein lipase and endothelial lipase, thereby reducing lipolysis and raising levels of circulating triglyceride-rich lipoproteins (TRL) and high-density lipoprotein (HDL), respectively (1, 2). Genetic and pharmacologic inactivation of ANGPTL3 in mice and humans leads to significant reductions in plasma lipids including total cholesterol and triglyceride (TG) (3, 4, 5). In fact, humans with complete genetic deficiency of ANGPTL3 exhibit familial combined hypolipidemia (FHBL2) and associated reductions in very low-density lipoprotein (VLDL) cholesterol, LDL, and HDL (6, 7, 8, 9, 10). This phenotype has been associated with a reduced risk of atherosclerotic plaque burden and related coronary events (11, 12). As a result, targeted inhibition of ANGPTL3 using a monoclonal antibody (evinacumab), an antisense oligonucleotide (ASO), and other pharmacologic strategies is an emerging therapeutic approach for treating refractory hyperlipidemia and potentially lowering cardiovascular disease risk.

Despite recent advances in the clinical applications of ANGPTL3 inhibition, the impact of ANGPTL3 deficiency on hepatic triglyceride-enriched lipoprotein assembly, secretion, and reuptake has not been fully characterized. These are important issues when considering the impact of genetic or pharmacologic inhibition of an important regulator of lipoprotein metabolism. For example, inhibition of either ApoB100 or microsomal triglyceride transfer protein—both key mediators of VLDL assembly and secretion—leads to hepatic steatosis (13, 14). In contrast, there are conflicting data regarding the metabolic impact of hepatic ANGPTL3 deficiency. Although treatment with the inhibitory ASO vupanorsen targeting ANGPTL3 production was recently associated with dose-dependent increases in hepatic steatosis that led to its discontinuation (15, 16), humans with complete genetic deficiency of ANGPTL3 have reduced VLDL-ApoB100 production without an increase in prevalence or severity of hepatic steatosis (5, 17, 18). The recent successful application of RNA interference against *ANGPTL3* in a phase 1 study also suggests that increased liver fat is not necessarily expected when inhibiting hepatic ANGPTL3 (15, 18, 19).

To address these questions and understand the potential intracellular role of hepatic ANGPTL3, we applied a combination of approaches to interrogate an in vitro hepatic model of complete ANGPTL3 deficiency using CRISPR/Cas9 targeting. We first set out to trace the fates of presecretory and postsecretory ApoB100 with an examination of lipoprotein assembly kinetics. We then compared the quantities of neutral lipid species present in wild type (*ANGPTL3*^*+/+*^) and *ANGPTL3*^*−/−*^ hepatic cells and profiled transcriptomic signatures of lipid metabolism in these cells. Lastly, we extended our analyses of ApoB100 secretion to *ANGPTL3^−/−^* cells deficient for LDLR while also examining their LDL uptake capacity. Collectively, our results point to an important intracellular role for ANGPTL3 that varies with LDLR functional status.

## Materials and methods

### Cell culture

HepG2 cells were obtained from American Type Culture Collection. Cells were maintained in Dulbecco’s modification of Eagle’s medium (DMEM) F-12 (Cat#11330-032, Gibco) containing GlutaMAX supplement (Cat# 35050061), 10% fetal bovine serum, 100 units ml^−1^ penicillin, and 100 g ml^−1^ streptomycin in 5% CO_2_ at 37°C. The medium was changed every 2–3 days.

### Generation of *ANGPTL3*^*−/−*^ and *LDLR*^*−/−*^ HepG2 cell lines

CRISPR/Cas9-engineered HepG2 lines were created by the Genome Engineering & Stem Cell Center (GESC) at Washington University in St. Louis. Briefly, synthetic genomic RNA targeting the *ANGPTL3* sequence (5′-AGTTCTTGGTGCTCTTGGCTNGG-3′) and LDL receptor (*LDLR*) sequence (5′-CGTGGTCGCTCTGGACACGGNGG-3′) were purchased from Integrated DNA Technologies, complexed with Cas9 recombinant protein, and transfected into wild type HepG2 cells. The transfected cells were then single-cell sorted into 96-well plates, and single cell clones were identified using next generation sequencing to analyze the target site region and select those harboring only out-of-frame alleles.

### ^35^S radiolabeling studies

Pulse-chase studies were performed according to the methods of Meex *et al.* (20) Briefly, cells were preincubated for 1 h at 37°C under 5% CO_2_ in serum-free methionine/cysteine-free DMEM (Cat# 21013024, Gibco), washed twice in Dulbecco’s phosphate buffered saline (DPBS; 200 mg/l KCl, 200 mg/l KH_2_PO_4_, 8,000 mg/l NaCl, 2,160 mg/l Na_2_HPO_4_-7H2O) without Ca^2+^ or Mg^2+^, and labeled for 15–20 min with methionine/cysteine-free DMEM supplemented with 200–300 μCi of EasyTag™ EXPRESS^35^S Protein Labeling Mix [^35^S] (Cat# NEG772002MC, Revvity) per ml of medium at 37°C, under 5% CO_2_. After labeling, the medium was removed, and cells were washed twice with DPBS. Cells were subsequently incubated with chase medium (serum-free DMEM replete with unlabeled methionine/cysteine). Chase period durations are indicated in figure legends. When the oleic acid (OA) stimulation of lipoprotein lipid loading was assessed, 0.2 mM or 0.6 mM OA complexed to BSA (molar ratio, 3:1) (Cat# O3008, Sigma-Aldrich) was provided throughout the course of the experiment. In some studies, 10 μM brefeldin A (Cat# B6542, Sigma-Aldrich), 100 nM bafilomycin (Cat #SML1661, Sigma-Aldrich) or 25 μM MG-132 (Sigma-Aldrich) was present throughout the course of the experiment as indicated in the figure legends.

### Immunoprecipitation of ApoB100

Following the chase period of the ^35^S radiolabeling experiments described above, cell culture medium was collected from cells. Cells were washed with Dulbecco’s Phosphate Buffered Saline (DPBS; 200 mg/l KCl, 200 mg/l KH_2_PO_4_, 8,000 mg/l NaCl, 2,160 mg/l Na_2_HPO_4_-7H2O) without Ca^2+^ or Mg^2+^ and lysed in cell lysis buffer (Cat#9803, Cell Signaling) freshly supplemented with protease inhibitor cocktail (Cat# P8340, Sigma-Aldrich). Lysed cells were gently shaken at 4°C for 5–10 min, transferred to an Eppendorf tube, and centrifuged at 14,000 rpm for 10 min in a table-top centrifuge at 4°C. To immune-precipitate ^35^S-ApoB100, conditioned medium or cell lysate was mixed with cell lysis buffer and anti-ApoB100 antibody (Cat#AB742) (in 1:100 dilution) or normal goat serum (Cat#NS02L) (Sigma Aldrich) as a negative isotype control. 10× lysis buffer was mixed with conditioned medium or lysate to a final concentration of 1× in the immunoprecipitation mixture. The mixture was incubated overnight with nutation at 4°C and subsequently incubated with Dynabeads Protein G beads (Cat#10003D, Thermo Fisher Scientific). The beads were blocked for 5–10 min with 2.5% BSA and washed 3–4 times before being added to each sample at a dilution of 1:25. The mixture was incubated with nutation for 30 min at room temperature (RT). The beads were pulled down using a magnet and washed 3–4 times with TBST. Where indicated, quantification of labeled ApoB100 was performed by SDS-PAGE, fluorography, and densitometry. In this case, the beads were run on a 2%–8% Criterion XT gel, which was then fixed, treated with EN3HANCE (Cat#6NE9701, PerkinElmer), dried, and exposed to radiographic film. Bands were excised, solubilized in SOLVABLE (Cat#6NE9100, PerkinElmer), and added to Ultima Gold scintillation fluid (Cat#L8286, Sigma-Aldrich) for counting. Otherwise, the beads were directly added to scintillation fluid and counted. Total protein synthesis was measured by determination of trichloroacetic acid (TCA) precipitable radioactivity in aliquots of conditioned medium and cell lysates. All data were normalized to total radiolabeled protein in media or lysate as indicated and expressed as percentage compared to the wild type controls.

### Subcellular fractionation

Subcellular fractionation was performed as described by Christova and Graham with modifications (21, 22). *ANGPTL3*^*+/+*^HepG2 and *ANGPTL3*^−^^*/*^^−^ HepG2 cells were seeded at a density of 8 × 10^6^/150 mm dish. The cells were cultured in DMEM/F-12 medium (Cat#11330-032, Gibco) supplemented with 10% fetal calf serum (FCS) and 100 IU ml^−1^ penicillin, 100 μg ml^−1^ streptomycin until 90% confluent. After reaching 90% confluence, the cells were washed with 5 ml DPBS (Cat#14190-136, Gibco), fasted in 15 ml serum-free DMEMF12 for 1 h, then switched to 15 ml DMEM/F-12 supplemented with 600 μM oleate BSA (Cat#O3008, Sigma-Aldrich). After being cultured for another 2 h, cells were washed with 5 ml ice-cold DPBS, gently scraped in 10 ml DPBS, and spun down at 150 *g* for 10 min. The cell pellet was suspended in 5.5 ml homogenization buffer STE (sucrose 0.25 M, Tris–HCL 10 mM and EDTA 1 mM, pH 7.4, 1× proteinase inhibitor cocktail). The cells were disrupted by 15 strokes in a Dounce homogenizer with pestle B. The homogenates were centrifuged at 1,000 *g* for 10 min in order to pellet nuclei and unbroken cells. The post nuclear supernatants were centrifuged in a Beckman MLA-130 at 42,000 rpm for 1 h at 4°C. The post nuclear pellet was resuspended in 0.5 ml of buffer STE with 10 strokes in a Dounce homogenizer. A gradient stock solution of 50% iodixanol was prepared by diluting Optiprep (Cat#D1556, Sigma-Aldrich) density gradient medium (60% W/V solution of iodixanol in water) with a buffer containing 0.25 M sucrose, Tris–HCL 60 mM, and EDTA 6 mM EDTA at a 5:1 ratio. The continuous gradients of 0%–30% Iodixanol were formed using the Biocomp Gradient Master Model 106 following instructions (Angle 79, Time 2:14, Speed 17 rpm) suggested by Biocomp. The resuspended post nuclear supernatants (0.5 ml) was loaded on top of the gradient and centrifuged in a Beckman SW41 rotor at 38,000 rpm (∼250,000 *g* max) for 3.5 h at 4°C. After centrifugation, sequential 0.5 ml fractions were collected from the top of the gradient. To concentrate the fractions and remove iodixanol, 300 μl from each fraction was diluted with 900 μl STE and centrifuged in a Beckman MLA-130 at 42,000 rpm (∼10,000 *g* max) for 1 h at 4°C. The vesicle pellet was resuspended into 40 μl buffer STE. Both concentrated fractions and unconcentrated fractions were stored at −80°C until further analysis. The protein in each concentrated fraction was separated in 4%–20% SDS-PAGE gradient gel (Cat#XP04122BOX, Invitrogen). The Apolipoprotein B (ApoB), beta-1,4-galactosyltransferase 1 (B4GalT1) and protein disulfide isomerase (an endoplasmic reticulum [ER] maker) in these fractions were analyzed by Western blot using the following specific antibodies: Millipore goat anti-human ApoB, Cat#AB742, 1:1,000; R&D System goat anti-human B4GalT1, Cat#AF3609, 1:1,000; and BD Transduction Laboratories™ purified mouse anti-PDI, Cat#610946, 1:500. The ApoB content in unconcentrated fractions was measured by human ApoB ELISA kit (Cat# 3715-1HP-2, Mabtech) following the manufacturer’s instructions.

### Targeted MS-Lipidomics

Following incubation with 200 μM OA overnight for 12 h, cells were suspended in PBS (5 × 10^6^ cells/ml). Modified Bligh-Dyer method was performed to extract TG, diacylglycerol (DAG), cholesterol, and cholesteryl ester (CE) from 50 μl of suspension. TG (17:0-17:0-17:0), DAG (21:0-21:0), d7-cholesterol, and d7-CE(18:2) were used as internal standards. Internal standards were added to the samples before extraction. Quality control (QC) samples were prepared by pooling the aliquots of the study samples and were used to monitor the instrument stability. Measurement of TG, DAG, cholesterol, and CE was performed with a Shimadzu 20A HPLC system coupled to an API4000 mass spectrometer (AB Sciex) operated in positive multiple reaction monitoring mode. Data processing was conducted with Analyst 1.6.3 (AB Sciex, https://sciex.com/support/software-support/software-downloads). The QC was injected between every three study samples. Only the lipid species with CV <15% in QC sample were reported. The relative quantification of lipids was provided, and the data were reported as the peak area ratios of the analytes to the corresponding internal standards. Upon observation of the mean-dependence of the variances calculated for quantified metabolites, data were log transformed to account for this heteroskedasticity. Differential metabolite analysis was performed in R using the limma-trend method (limma v3.16, https://bioconductor.org/packages/release/bioc/html/limma.html) (23). Pathway analyses were conducted using LION/web (v. 2020.07.14, http://www.lipidontology.com) lipid ontology and enrichment analysis (24).

### ^3^H and ^14^C radiolabeling studies

*ANGPTL3*^*+/+*^ and *ANGPTL3*^*−/−*^ HepG2 cells were seeded at 3–4 × 10^5^ per 35 mm dish and cultured overnight in DMEM/F12 supplemented with 10% FCS and penicillin-streptomycin 100 U ml^−1^. After washing once with PBS, cells were cultured in serum-free medium for 1 h and then pulse labeled in serum-free medium supplemented with 1 μCi ml^−1^ [^3^H]oleate (#ART0198, American Radiolabeled Chemicals) or [^14^C]acetate (#ARC173A, American Radiolabeled Chemicals) and 200 μM oleate BSA (#O3008, Sigma-Aldrich) for 4 h, before switching to unlabeled medium with 200 μM oleate BSA. Cells and medium were collected at 0, 4, and 16 h. Lipids were extracted with Folch reagent (chloroform: methanol 2:1). Lipid classes were resolved using thin layer chromatography (hexane: diethyl ether: acetic acid, 70:30:1) and TG bands identified based on the migration of authentic standards. TG bands were scraped and radioactivity counted in 5 ml Ultima Gold scintillation fluid (Cat#L8286, Sigma-Aldrich) All data were normalized to total cellular protein and expressed as percentage compared to the wild type controls.

### Palmitate oxidation assay

Measurements of fatty acid oxidation were performed according to the tritiated water release method of Djouadi *et al.* (25). Briefly, 3 × 10^5^ cells were plated per well of a 12 well dish. Cells were washed three times with PBS—minus Ca^2+^ and Mg^2+^, but containing 200 μl of (9,10(n)-^3^H) palmitic acid (#NET-043, Revvity) bound to fatty-acid free albumin (125 μM final concentration) and supplemented with 1 mM L-carnitine (#C0158, Sigma-Aldrich)—added to each well. Incubation was carried out for 2 h with gentle agitation at 37°C. In some experiments, 50 μM WY-14643 (positive control) or 10 μM etomoxir (negative control) were added to the incubation media. After incubation, the mixture was removed and added to a tube containing 200 μl of cold 10% TCA. Tubes were centrifuged 10 min at 2,200 *g* at 4°C. Three hundred microliters of supernatant was removed, mixed with 55 μl of 6 M NaOH, and applied to ion-exchange resin (DOWEX 1 x 2-400 #217395, Sigma-Aldrich). Columns were eluted with 1.7 ml distilled water, and the eluates were counted. Measurements were normalized to cell protein count determined by bicinchoninic acid (BCA).

### Bulk RNAseq

Following incubation with 200 μM OA (or vehicle control) overnight for 12 h, total RNA was purified from cells using the RNeasy Mini Kit (#NC9677589, Qiagen). High-Capacity complementary DNA (cDNA) Reverse Transcription Kit (#4368814, Thermo Fisher Scientific) with random priming was used to obtain full-length reverse transcripts. Total RNA integrity was determined using Agilent Bioanalyzer or 4200 TapeStation. Library preparation was performed with 5–10 μg of total RNA with a Bioanalyzer RNA integrity number score greater than 8.0. Ribosomal RNA was removed by poly-A selection using Oligo-dT beads (mRNA Direct kit, #610.11, Life Technologies). mRNA was then fragmented in reverse transcriptase buffer and heated to 94° for 8 min mRNA was reverse transcribed to yield cDNA using SuperScript III RT enzyme (#18080093, Life Technologies, per manufacturer's instructions) and random hexamers. A second strand reaction was performed to yield ds-cDNA. cDNA was blunt ended, had an A base added to the 3′ ends, and then had Illumina sequencing adapters ligated to the ends. Ligated fragments were then amplified for 12–15 cycles using primers incorporating unique dual index tags. Fragments were sequenced on an Illumina NovaSeq-6000 using paired end reads extending 150 bases. Basecalls and demultiplexing were performed with Illumina’s bcl2fastq (https://support.illumina.com/sequencing/sequencing_software/bcl2fastq-conversion-software.html) software and a custom python demultiplexing program with a maximum of one mismatch in the indexing read. Read quality was assessed with Fastqc. Adapter sequences and low quality bases were trimmed using fastp using –detect_adapter_for_pe (26). Reads were aligned to the human genome (GRCh38) using STAR (27) with parameters: '--readFilesCommand zcat --outStd BAM_SortedByCoordinate --outSAMtype BAM SortedByCoordinate --alignMatesGapMax 1000000 --outFilterMismatchNmax 999 --alignIntronMax 1000000 --alignSplicedMateMapLmin 3 --alignSJoverhangMin 8 --alignSJDBoverhangMin 1 --outFilterMismatchNoverReadLmax 0.04 --outSAMunmapped Within KeepPairs --outSAMattributes All --alignIntronMin 20 --outFilterIntronMotifs RemoveNoncanonicalUnannotated --scoreGapNoncan -14 --outSJfilterReads Unique --outFilterMultimapNmax 10’. Alignments were then assigned to genic features using featurecounts from the subread package (28) with parameters '-p -g gene_id -s 2 -Q 5 --extraAttributes gene_type,gene_name'. Differential gene expression analysis was performed using the R package DESeq2 (1.30.1, https://bioconductor.org/packages/release/bioc/html/DESeq2.html). Gene expression was modeled using genotype and treatment as dependent variables along with their interaction (∼genotype+treatment+genotype:treatment). Gene set enrichment analysis was performed using the R package clusterProfiler (3.18.1, https://bioconductor.org/packages/release/bioc/html/clusterProfiler.html) (29). RNAseq data processing code (.fastq>counts) is available at https://doi.org/10.5281/zenodo.7469624 and https://doi.org/10.5281/zenodo.7469921.

### Ultracentrifugation for TG extraction and ApoB100 measurement

*ANGPTL3*^*+/+*^ and *ANGPTL3*^*−/−*^ HepG2 cells were seeded at 8 × 10^5^ in T25 flasks and cultured overnight in DMEM F12 supplemented with 10% FCS and penicillin-streptomycin (100 U ml^−1^). After washing once with PBS, cells were cultured in 2.5 ml DMEM/F-12 containing 200 μM oleate BSA (#O3008, Sigma-Aldrich) for 16 h. The medium was collected and spun down at 2,000 rpm for 5 min to remove the cell debris. The supernatant was collected and its density was adjusted by sodium bromide to 1.21 g ml^−1^. After spinning down at 100,000 rpm (606,800 *g* max) for 4 h at 10°C in a Beckman rotor MLA-130, the top one-third was collected as lipoprotein-rich part (LPR). Cells were harvested into 800 μl M-PER™ mammalian protein extraction reagent (#78501, Thermo Fisher Scientific) following manufacturer’s instruction, and protein content was determined by detergent compatible protein assay reagents (#5000113, #5000114, and #5000115, Bio-Rad). Triglycerides in LPR were extracted with Folch reagent and quantitated by Triglyceride M reagents (#992-02892, #998-02992, Wako). ApoB in LPR was quantitated by Mabtech ApoB ELISA kit. Data are presented as the ratio of ApoB100:TG after normalization to cellular protein content.

### BODIPY-LDL uptake assays

Cells were treated with LDL from human plasma BODIPY™ FL complex (Cat# L3483, Thermo Fisher Scientific) according to the manufacturer’s protocol. Briefly, cells were serum starved in FluoroBrite™ DMEM (Cat# A1896701, Thermo Fisher Scientific) for 12–24 h before incubating with 10 μg/ml labeled LDL for 2–4 h. Cells were washed three times in DPBS + 0.3% BSA. For flow cytometric analysis, cells were dissociated using CellStripper (Cat# 25-056-CI, Corning), and cell preps were centrifuged at 1,700 rpm for 3 min at RT. Cells were washed twice at 2,000 rpm for 2 min at RT before fixation using eBioscience FoxP3 Staining Buffer Kit (Cat# 00-5523-00, Thermo Fisher Scientific) in the dark at RT. Flow cytometric analyses were performed using LSRFortessa instrument (BD Biosciences) and FlowJo (https://www.flowjo.com) software (Tree Star Inc).

### Fluorescent microscopy

Cells were grown on sterile glass coverslips under culture conditions described above unless otherwise noted. Cells were fixed using 4% paraformaldehyde and permeabilized with 0.5% TritonX-100 for 10 min. Cells were then blocked with PBS containing 5% donkey serum (#S-3000, Vector Laboratories) with 0.5% TritonX-100 for 1 h at room temperature. Cells were then incubated with primary antibodies overnight at 4°C and with the corresponding secondary antibodies for 1 h at room temperature. Cells were mounted on slides using ProLong Diamond Antifade with DAPI (Cat# P36961, Thermo Fisher Scientific) in preparation for l scanning on a Zeiss LSM 700 laser scanning microscope and analysis using the Zeiss Zen (v.3.7, https://www.zeiss.com/microscopy/en/products/software/zeiss-zen.html) software. Corrected total cell fluorescence was computed using ImageJ (v1.53, https://imagej.net/ij/).

### Immunoblot assays

Samples were lysed with cell lysis buffer (#9803, Cell Signaling Technologies) containing a protease inhibitor cocktail (Cat# P8340, Sigma-Aldrich). Protein immunoblots were performed by the following standard techniques. Briefly, protein content was determined using a BCA assay with BSA standards (#23225, Pierce BCA Protein Assay Kit). Cell lysates were reduced with DTT in lithium dodecyl sulfate sample buffer (#NP0007, Invitrogen). Equal protein amounts were added to polyacrylamide gels (#4561086, BioRad) and electrophoresed prior to transferring to a nitrocellulose or polyvinylidene fluoride membrane (#1620260, BioRad). Membranes were blocked in Intercept TBS Blocking Buffer (#927-60001, LI-COR) for 1 h. Indicated primary antibodies were incubated with the preblocked membranes overnight at 4°C. Membranes were washed with Tris-buffered saline with Tween 20, probed with fluorescent secondary antibodies, and imaged. β-actin or β-tubulin served as a loading control. In experiments where immunoprecipitated ApoB100 was blotted with anti-Grp78 (Stressgen Cat# SPA-726), a concentration of 1:2000 was used.

### Quantitative real-time PCR

Gene expression was quantified by quantitative real-time PCR. RNA was isolated from QIAshredder (#79656)-homogenized cells using RNeasy Mini Kit (#74106) according to the manufacturer’s protocol (all purchased from Qiagen). cDNA was synthesized with High Capacity cDNA Reverse Transcript Kit (#4368814, Applied Biosystems). Real-time PCR was performed using PowerUp SYBR (#A25742, Applied Biosystems) assays. Cycle threshold (Ct values) were normalized to β-actin and showed as expression relative to control.

### Statistical analysis

Unless otherwise stated, cellular assays were analyzed by an unpaired, two-tailed, Student’s *t* test. Unless otherwise stated, statistical analyses were performed with GraphPad Prism (https://www.graphpad.com) with quantitative results displayed as means ± SEM.

## Results

### ANGPTL3 deficiency leads to decreased secretion and enhanced degradation of ApoB100 in HepG2 cells

**Fig. 1:**
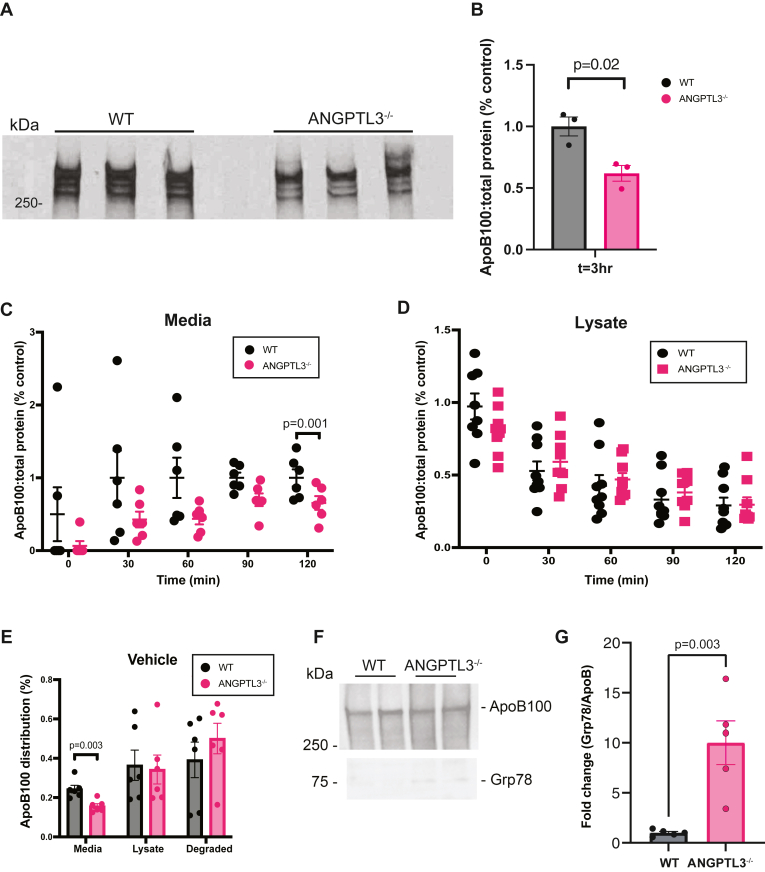
ANGPTL3 deficiency decreases ApoB100 secretion and promotes chaperone-mediated degradation of ApoB100 in HepG2 cells. A: Cells were pulsed (150 μCi/ml) for 20 min in media supplemented with 200 μM oleic acid and chased with cold media replete with cysteine and methionine (also containing 200 μM oleic acid). ApoB100 was pulled down from media collected 3 h after the initial 20 min pulse period. Samples were run on a 3%–7% gel, fixed, treated with E3HANCE, dried, and exposed to radiographic film. n = 3 wells per genotype. B: Radiolabeled ApoB100 immunoprecipitated from media collected after 3 h. Counts were normalized to total TCA precipitable protein. n = 3 wells per genotype. *P*-value as indicated by unpaired Student’s *t* test. C: Radiolabeled ApoB100 immunoprecipitated from media collected at the indicated time points was counted and normalized to total TCA precipitable protein. n = 6 wells per group per genotype. *P*-value as indicated by ordinary two-way ANOVA with Sidak’s correction for multiple comparisons. D: Radiolabeled ApoB100 immunoprecipitated from lysates collected at the indicated time points was counted and normalized to total TCA precipitable protein. n = 6 wells per group per genotype. E: Radiolabeled ApoB was immunoprecipitated from media and lysates collected 3 h after the initial 20 min pulse period. ApoB distribution at 3 h was calculated as a percentage based on ApoB100 present after the initial 20 min pulse period. Counts were normalized to total TCA precipitable protein. n = 6 wells per genotype. *P*-values as determined by two-way ANOVA with Sidak correction performed on full dataset presented in Fig. 6. F: ApoB100 immunoprecipitated from unlabeled lysates and blotted for Grp78. G: Quantification of fold-change of Grp78 normalized to ApoB100. n = 5 samples per group. *P*-value as indicated by unpaired Student’s *t* test. ANGPTL3, angiopoietin-like protein 3; ApoB100, apolipoprotein B100; TCA, trichloroacetic acid.

We used CRISPR-Cas9 to generate independent clones where ANGPTL3 was stably “knocked out” in HepG2 cells (supplemental Fig. S1). With this model, we first asked if ANGPTL3 deficiency affected the secretion of ApoB100. Despite no baseline difference in *APOB* transcript abundance (supplemental Fig. S2), we observed *ANGPTL3*^*−/−*^ cells exhibited significantly decreased secretion of ApoB100 into oleic acid-containing cell culture media 3 h after an initial radiolabeling pulse of 20 min (Fig. 1A, B). We examined ApoB100 secretion at multiple time points and observed decreased ApoB100 secretion across the time course (Fig. 1C), which was replicated in independent experiments using a separate ANGPTL3 knockout clone (supplemental Fig. S3).

Despite decreased ApoB100 secretion, we observed that ApoB100 immunoprecipitated from cell lysates did not significantly differ between *ANGPTL3^+/+^* and *ANGPTL3*^*−/−*^ cells across multiple time points (Fig. 1D). Based on this observation, we asked if the decreased ApoB100 secretion could be attributed to enhanced degradation in *ANGPTL3*^*−/−*^ cells. To address this question, we used pulse-chase radiolabeling studies to determine the percentage of ApoB100 present in media and lysates. ApoB100 secretion was again reduced in *ANGPTL3*^*−/−*^ cells and we observed a trend toward increased ApoB100 degradation (Fig. 1E). We also observed an increase in Grp78 chaperone binding to unlabeled ApoB100 immunoprecipitated from *ANGPTL3*^*−/−*^ cell lysates, suggesting that proteasomal or other degradative pathways may be involved in the presecretory defects observed in ApoB100 secretion.

**Fig. 2:**
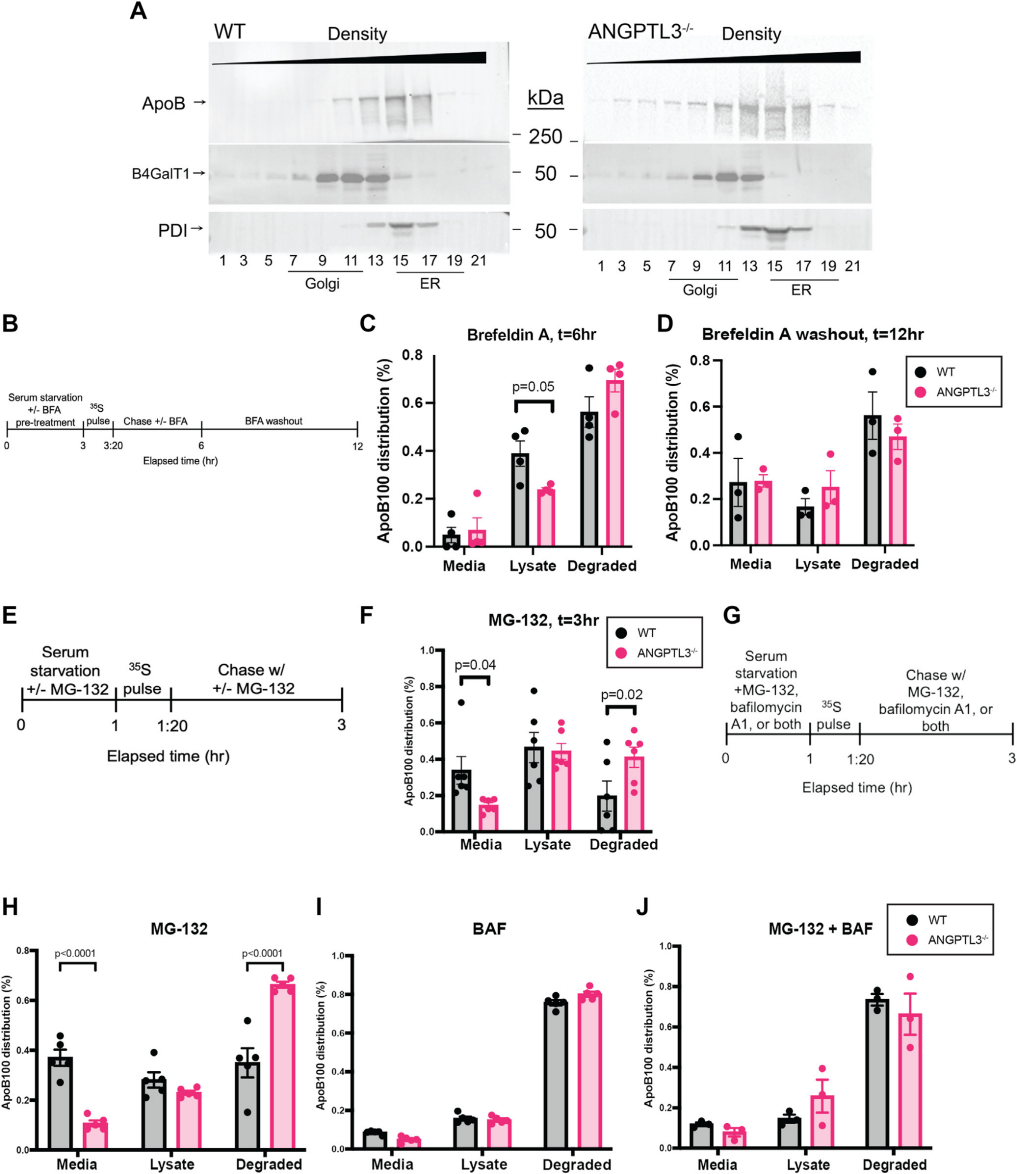
ApoB100 degradation is enhanced in a pre-Golgi compartment in ANGPTL3-deficient HepG2 cells. A: Cells were treated with 600 μM oleic acid, homogenized, and fractionated by ultracentrifugation across a continuous iodixanol gradient. Proteins in concentrated fractions were separated on a 4%–20% SDS-PAGE gradient gel. B: Cells were pulsed (150 μCi/ml) for 20 min in media supplemented with 600 μM oleic acid and 10 μM brefeldin A. Cells were then chased with cold media replete with cysteine and methionine (also containing 600 μM oleic acid and 10 μM brefeldin A). ApoB100 was immunoprecipitated from media and lysates collected 6 h after the beginning of the experiment and after a washout period ending 12 h after the beginning of the experiment. C: ApoB distribution at t = 6 was calculated as a percentage based on ApoB100 present at t = 3:20. n = 4 wells per group per genotype. *P*-value as determined by two-way ANOVA with Sidak correction performed on full dataset presented in Fig. 6. D: ApoB distribution at t = 12 was calculated as a percentage based on ApoB100 present at t = 3:20. n = 4 wells per group per genotype. *P*-value as indicated by unpaired Student’s *t* test. E: Cells were pulsed (150 μCi/ml) for 20 min in media supplemented with 600 μM oleic acid and 25 μM MG-132 where indicated. Cells were then chased with cold media replete with cysteine and methionine (also containing 600 μM oleic acid and 25 μM MG-132). F: ApoB100 was immunoprecipitated from lysates collected 3 h after the beginning of the experiment. ApoB distribution was calculated as a percentage based on ApoB100 present at t = 1:20. Counts were normalized to total TCA precipitable protein. n = 6 wells per genotype. *P*-values as determined by two-way ANOVA with Sidak correction performed on full dataset presented in Fig. 6. G: Cells were pulsed (150 μCi/ml) for 20 min in media supplemented with 600 μM oleic acid and 100 nM bafilomycin A1 in the presence and absence of 25 μM MG-132. Cells were then chased with cold media replete with cysteine and methionine (also containing 600 μM oleic acid and 100 nM bafilomycin A1 ± MG-132). ApoB100 was immunoprecipitated from media and lysates collected 6 h after the beginning of the experiment and after a washout period ending 12 h after the beginning of the experiment. H: ApoB distribution at t = 3 was calculated as a percentage based on ApoB100 present at t = 1:20. n = 5 wells per group per genotype. *P*-value as indicated by unpaired Student’s *t* test. I: ApoB distribution at t = 3 was calculated as a percentage based on ApoB100 present at t = 1:20. n = 5 wells per group per genotype. J: ApoB distribution at t = 3 was calculated as a percentage based on ApoB100 present at t = 1:20. n = 3 wells per group per genotype. ANGPTL3, angiopoietin-like protein 3; ApoB100, apolipoprotein B100.

We next wanted to propose a site of action within the secretory pathway where ApoB100 degradation could differ between *ANGPTL3*^*+/+*^ and *ANGPTL3*^*−/−*^ cells. To address this question, we first performed subcellular fractionation on unlabeled, untreated lysates. *ANGPTL3*^*−/−*^ cells exhibited a shift in ApoB100 toward lower-density Golgi fractions (Fig. 2A), suggesting that at least a portion of intracellular ApoB100 may be retained in a presecretory compartment. To pinpoint whether the degradative capacity of *ANGPTL3^+/+^* and ANGPTL3-deficient cells differed in a pre- or post-Golgi compartment, we performed pulse-chase radiolabeling studies using cells treated with brefeldin A to block ER-to-Golgi transit and inhibit protein secretion (Fig. 2B). Consistent with a pre-Golgi site of action, ANGPTL3-deficient cells treated with brefeldin A showed a statistically significant decrease in ApoB100 immunoprecipitated from lysates as well as a concomitant trend toward increased ApoB100 degradation (Fig. 2C); this effect was reversed with washout of brefeldin A (Fig. 2D). However, when we attempted to rescue the secretion defect by inhibiting the proteasome with MG-132 treatment, we observed a persistent secretion defect and a significant increase in the proportion of ApoB100 degraded by ANGPTL3-deficient cells (Fig. 2E, F and supplemental Fig. S4). In contrast, inhibition of the lysosomal degradative pathway using bafilomycin A1, both in the presence and absence of MG-132, restored ApoB100 secretion to wild type levels (Fig. 2G–J). Taken together, these data suggest the TRL secretion defect observed in ANGPTL3-deficient cells is the result of increased ApoB100 degradation by a predominantly lysosomal, pre-Golgi mechanism.

### ANGPTL3 deficiency leads to adaptive changes in hepatic lipid metabolism

**Fig. 3:**
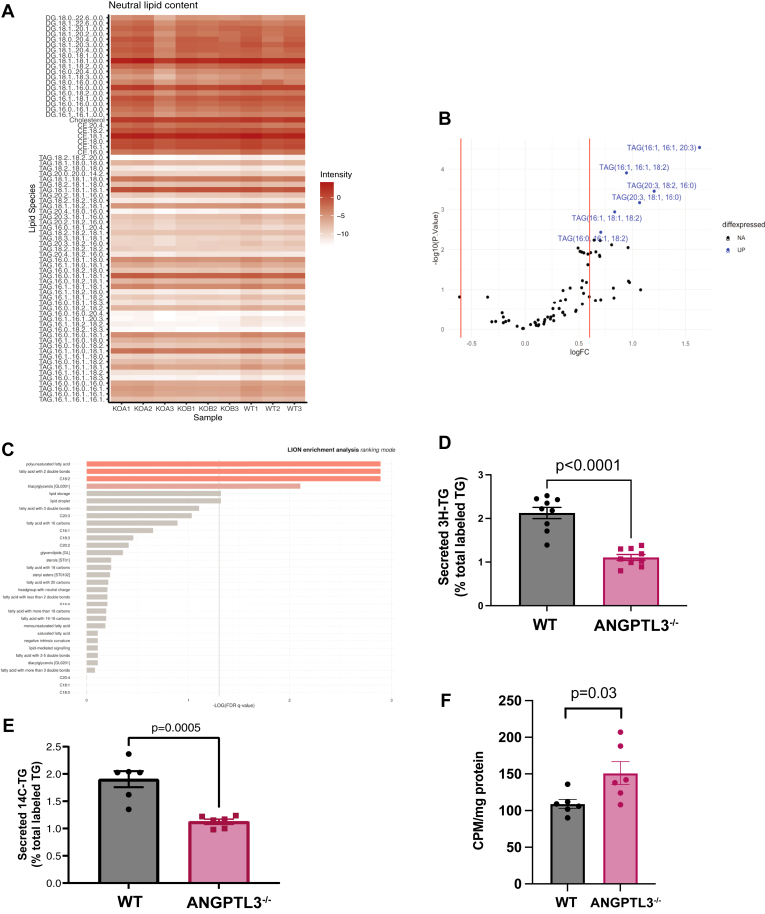
ANGPTL3 deficiency does not lead to neutral lipid accumulation in HepG2 cells. A: wild type and ANGPTL3-deficient HepG2 cells (two clones) were treated for 12 h with 200 μM OA prior to targeted lipidomic evaluation. Data were log transformed to account for heteroskedasticity; differential metabolite analysis was performed in R using the limma-trend method (limma v3.16) Data expressed as log2FC. n = 3 pools of 2 dishes per genotype. B: Volcano plot of mass spec lipidomics experiment. Differentially expressed (“diffexpressed”) lipid species shown in blue are upregulated in wild type cells relative to KO. C: Results of Lipid Ontology (LION) enrichment analysis performed in ranking mode. Similar results were obtained in target list mode. D: Cells were cultured in serum free medium for 1 h before labeling with media containing 1 μCi/ml [3H]-oleate and 200 μM oleate BSA. Cells were labeled for 4 h before switching to unlabeled medium with 200 μM oleate BSA. Medium was collected at 0 and 4 h. Lipids were extracted with Folch reagent. Lipid classes were resolved using thin layer chromatography. TG bands were scraped and counted. All data were normalized to total cellular protein and expressed as percentage of total labeled TG. n = 6 wells per genotype. E: Cells were cultured in serum-free medium for 1 h before labeling with media containing 1 μCi/ml [14C]-acetate and 200 μM oleate BSA. Cells were labeled for 4 h before switching to unlabeled medium with 200 μM oleate BSA. Medium was collected at 0 and 4 h. Lipids were extracted with Folch reagent. Lipid classes were resolved using thin layer chromatography. TG bands were scraped and counted. All data were normalized to total cellular protein and expressed as percentage of total labeled TG. n = 6 wells per genotype. F: Cells were labeled for 2 h with 125 μM ^3^H palmitate (supplemented with 1mM L-carnitine). Labeling media was subjected to TCA precipitation and NaOH neutralization prior to application to DOWEX ion exchange resin. Columns were eluted with 1.7 ml water, and the eluates were counted. n = 6 wells per genotype. ANGPTL3, angiopoietin-like protein 3; BSA, bovine serum albumin; OA, oleic acid; TCA, trichloroacetic acid; TG, triglyceride.

Our observations regarding impaired ApoB100 assembly and secretion in *ANGPTL3*^*−/−*^ cells led us to ask whether these cells accumulate neutral lipids at steady state. To address this question, we performed mass spectrometry-based targeted lipidomic analysis to quantify neutral lipids present in *ANGPTL3^+/+^* and *ANGPTL3*^*−/−*^ cells treated with 200 μM OA (Fig. 3A). While 6 of 47 TG species were significantly decreased in *ANGPTL3*^*−/−*^ cells relative to wild type controls (Fig. 3B, C), ANGPTL3 deficiency did not lead to hepatic accumulation of neutral lipids at steady state.

To further functionally characterize the lipid metabolism of ANGPTL3-deficient HepG2 cells, we measured the rate of newly synthesized TG secretion into the cell culture media using radiolabeled substrates to quantify TG synthesis and lipogenesis. Neutral lipids were labeled to steady-state with either [^3^H]-labeled oleate (Fig. 3D and supplemental Fig. S5) or [^14^C]-labeled acetate (Fig. 3E), which demonstrated comparable accumulation of newly synthesized TG by genotype (supplemental Fig. S5). However, we observed significant reductions in secretion of both [^3^H]-labeled and [^14^C]-labeled TGs (Fig. 3D, E) from *ANGPTL3*^*−/−*^ cells relative to wild type controls, consistent with decreased secretion of newly synthesized TRLs into the media. Using the ratio of ApoB100 to triglyceride as a rough approximation of particle size, we did not observe significant differences between the two genotypes (supplemental Fig. S6). This defect in triglyceride secretion without accompanying lipid accumulation led us to ask if lipid catabolism was increased. Accordingly, we measured fatty acid oxidation by incubating cells with ^3^H-palmitate and quantifying tritiated water release and observed a statistically significant increase in *ANGPTL3*^−/−^ cells, consistent with increased fatty acid oxidation (Fig. 3F).

**Fig. 4:**
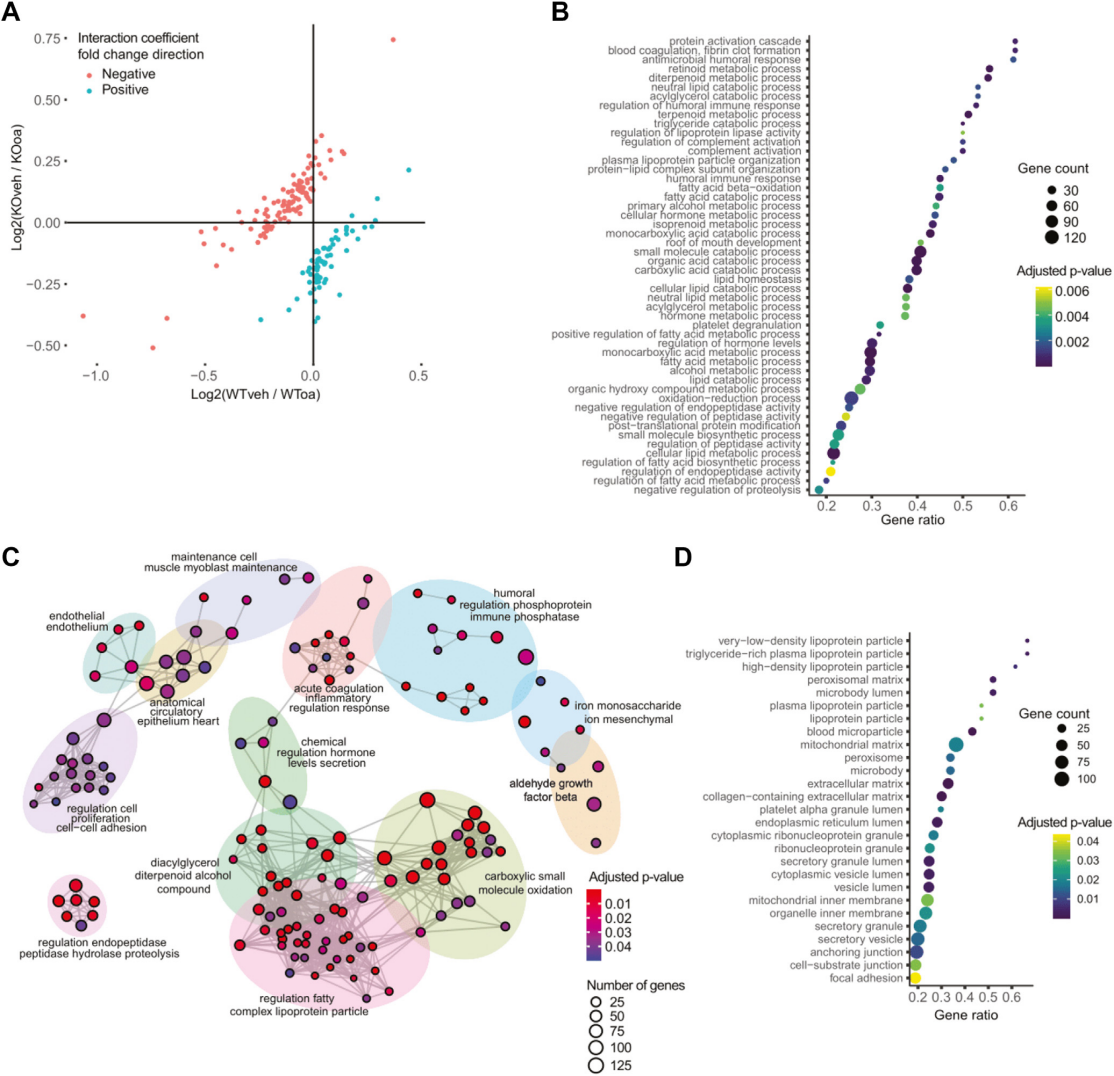
ANGPTL3-deficient HepG2 cells show transcriptional evidence of increased lipid catabolism. Wild type and ANGPTL3-deficient HepG2 cells were treated with vehicle control (FA-free BSA) or 200 μM oleic acid overnight prior to RNA extraction and bulk RNA sequencing. n = 3 wells per genotype. A: Differential gene expression analysis was performed using DESeq2, and gene expression was modeled using genotype and treatment as dependent variables along with their interaction (∼genotype + treatment + genotype:treatment). Significant interaction coefficients (adjusted *P*-value < 0.05) and the direction of their fold change relative to wild type are plotted here. B: Top 50 upregulated GO biological process (BP) terms as determined by GSEA. Upregulation reflects increased gene expression in ANGPTL3-deficient cells over wild type. Gene count indicates number of genes in the significant differentially expressed gene list associated with the GO term. Gene ratio reflects the extent to which genes in the set are over-represented. C: Functional grouping network diagram generated by emapplot_cluster for results of GSEA GO BP analysis in (B). D: Top 25 enriched GO cellular compartment (CC) terms as determined by GSEA and represented as in (B). ANGPTL3, angiopoietin-like protein 3; BSA, bovine serum albumin; GO, Gene Ontology; GSEA, gene set enrichment analysis.

**Fig. 5:**
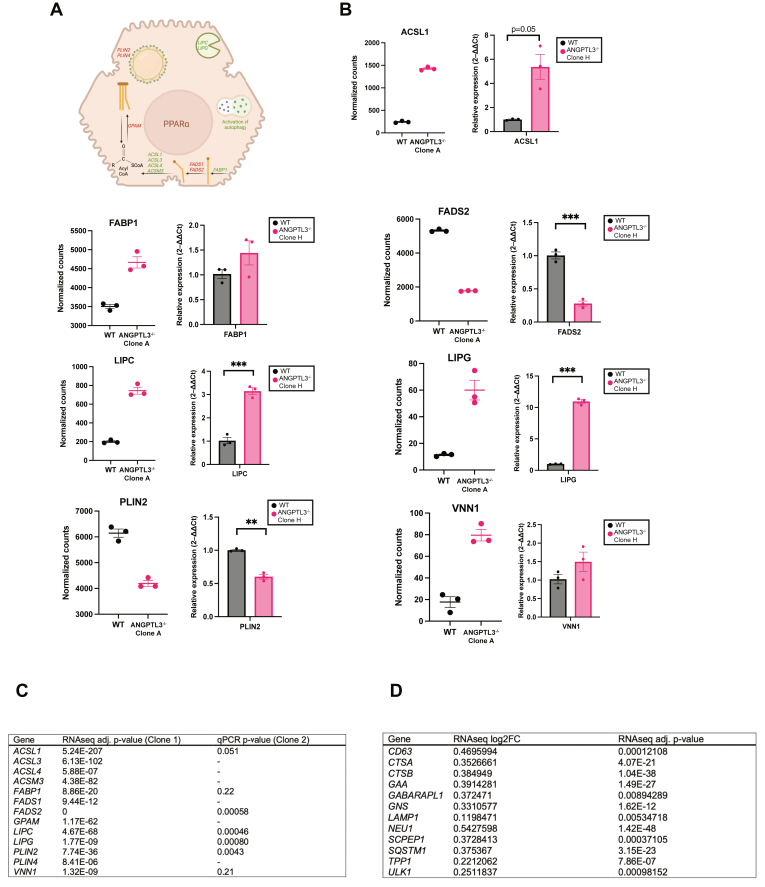
Key PPARα target genes are differentially expressed between wild type and ANGPTL3^−/−^ HepG2 cells. Wild type and ANGPTL3-deficient HepG2 cells were treated with vehicle control (FA-free bovine serum albumin) or 200 μM oleic acid overnight prior to RNA extraction and bulk RNA sequencing. n = 3 wells per genotype. A: Genes listed on diagram showed strong differential expression that was validated in a second ANGPTL3 knockout HepG2 clone by qPCR. B: For each gene, the RNA sequencing result from the 200 μM oleic acid treatment condition is demonstrated in the left panel. The right panel shows validation in a second *ANGPTL3*^−/−^ HepG2 clone by qPCR. C: Table of *P*-values from (B). D: Table of autophagy-related genes upregulated in ANGPTL3-deficient HepG2 cells. ANGPTL3, angiopoietin-like protein 3; PPARα, peroxisome proliferator-activated receptor α; qPCR, quantitative real-time PCR.

To explore if compensatory changes in hepatic lipid metabolism might account for the lack of neutral lipid accumulation in ANGPTL3-deficient cells, we performed RNA sequencing to assess differential gene expression between genotypes (Fig. 4A and supplemental Fig. S7). To assess differences in response to treatment between genotypes, we modeled gene expression as ∼genotype + treatment + genotype:treatment and focused on genes with significant interaction coefficients (Fig. 4A). This analysis found that ANGPTL3-deficient cells exhibited upregulation of several lipid metabolism genes relative to wild type controls (Fig. 4B, C). Furthermore, the transcripts of these genes encode protein products that are predicted to localize to subcellular compartments related to lipoprotein assembly and lipid metabolism (Fig. 4D). We also noted many of these differentially expressed transcripts were targets of peroxisome proliferator-activated receptor α (PPARα) (Fig. 5A), a known regulator of lipid metabolism, including fatty acid oxidation (Fig. 5B, C) and an activator of hepatic autophagy (Fig. 5D). These findings provide further evidence of compensatory transcriptional changes in hepatic lipid metabolism favoring a combination of decreased lipogenesis and increased lipid catabolism that mitigate accumulation of neutral lipids in the setting of hepatic ANGPTL3 deficiency.

### ApoB100 degradation is enhanced in a post-Golgi compartment in ANGPTL3-deficient HepG2 cells lacking LDLR

**Fig. 6:**
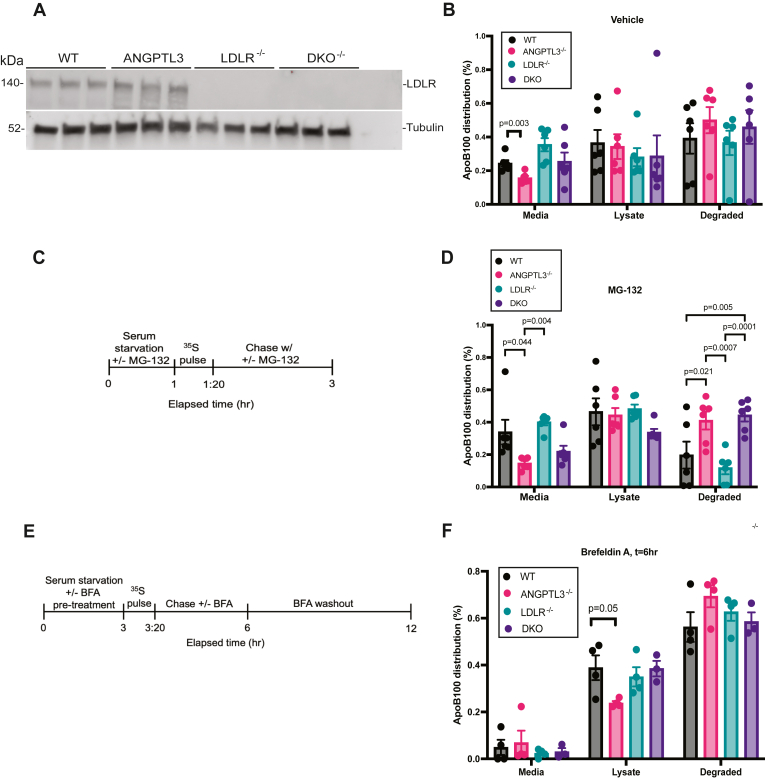
ApoB100 degradation is enhanced in a post-Golgi compartment in ANGPTL3-deficient HepG2 cells lacking LDLR. A: Validation of LDLR knockout in CRISPR-edited HepG2 cells. DKO = *ANGPTL3*^*−/−*^*;LDLR*^*−/−*^ HepG2 cells. B: Cells were pulsed (150 μCi/ml) for 20 min in media supplemented with 600 μM oleic acid. Cells were then chased with cold media replete with cysteine and methionine (also containing oleic acid). ApoB100 was immunoprecipitated from media and lysates collected 3 h after the initial 20 min pulse period. ApoB distribution was calculated as a percentage based on ApoB100 present at t = 1:20. Counts were normalized to total TCA precipitable protein. n = 6 wells per genotype. *P*-values as indicated by two-way ANOVA with Sidak correction. C: Cells were pulsed (150 μCi/ml) for 20 min in media supplemented with 600 μM oleic acid and 25 μM MG-132. Cells were then chased with cold media replete with cysteine and methionine (also containing oleic acid and 25 μM MG-132). D: ApoB100 was immunoprecipitated from media and lysates collected 3 h after the initial 20 min pulse period. ApoB distribution was calculated as a percentage based on ApoB100 present at t = 1:20. Counts were normalized to total TCA precipitable protein. n = 6 wells per genotype. *P*-values as indicated by two-way ANOVA with Sidak correction. E: Cells were pulsed (150 μCi/ml) for 20 min in media supplemented with 600 μM oleic acid and 10 μM brefeldin A. Cells were then chased with cold media replete with cysteine and methionine (also containing 600 μM oleic acid and 10 μM brefeldin A). F: ApoB100 was immunoprecipitated from media and lysates collected 6 h after the beginning of the experiment. ApoB distribution was calculated as a percentage based on ApoB100 present at t = 3:20. n = 4 wells per group per genotype. ANGPTL3, angiopoietin-like protein 3; ApoB100, apolipoprotein B100; LDLR, low-density lipoprotein receptor; TCA, trichloroacetic acid.

Previous studies have demonstrated that inhibiting ANGPTL3 leads to decreased plasma LDL cholesterol in an LDLR-independent manner (30, 31), motivating us to ask whether the same ApoB100 degradative mechanisms examined above were present in ANGPTL3-deficient cells lacking the LDL receptor. To accomplish this, we used CRISPR/Cas9 to generate *LDLR*^*−/−*^ HepG2 cells in the presence and absence of *ANGPTL3* (Fig. 6A and supplemental Fig. S8). We performed the same battery of testing using pulse-chase radiolabeling studies to determine ApoB100 distribution profiles for *LDLR*^*−/−*^ and *ANGPTL3*^*−/−*^*;LDLR*^*−/−*^ cells at the same time as the profiles generated for *ANGPTL3^+/+^* and *ANGPTL3*^*−/−*^ cells in Fig. 2 above. At baseline, in contrast to the decreased secretion by *ANGPTL3*^*−/−*^ cells expressing LDLR, we did not observe differences in ApoB100 immunoprecipitated from the media or lysates of *ANGPTL3*^*−/−*^ cells lacking the LDLR. Furthermore, we also did not observe differences in calculated ApoB100 degradation in these *ANGPTL3*^*−/−*^*;LDLR*^*−/−*^ cells at baseline. (Fig. 6B). However, when we performed the MG-132 inhibition study, we observed a decrease in media ApoB100 accompanied by increased degradation in *ANGPTL3*^*−/−*^*;LDLR*^*−/−*^ cells (Fig. 6C, D) but we did not observe differences in the degradation of ApoB100 when we repeated the brefeldin A pulse-chase experiment (Fig. 6E, F). Taken together, these data suggest a late- and/or postsecretory mechanism of ApoB100 regulation that appears distinct from the early secretory regulation of ApoB100 degradation exhibited by ANGPTL3-deficient cells expressing functional LDLR (32).

### ANGPTL3 deficiency restores LDL uptake in the absence of LDLR

**Fig. 7:**
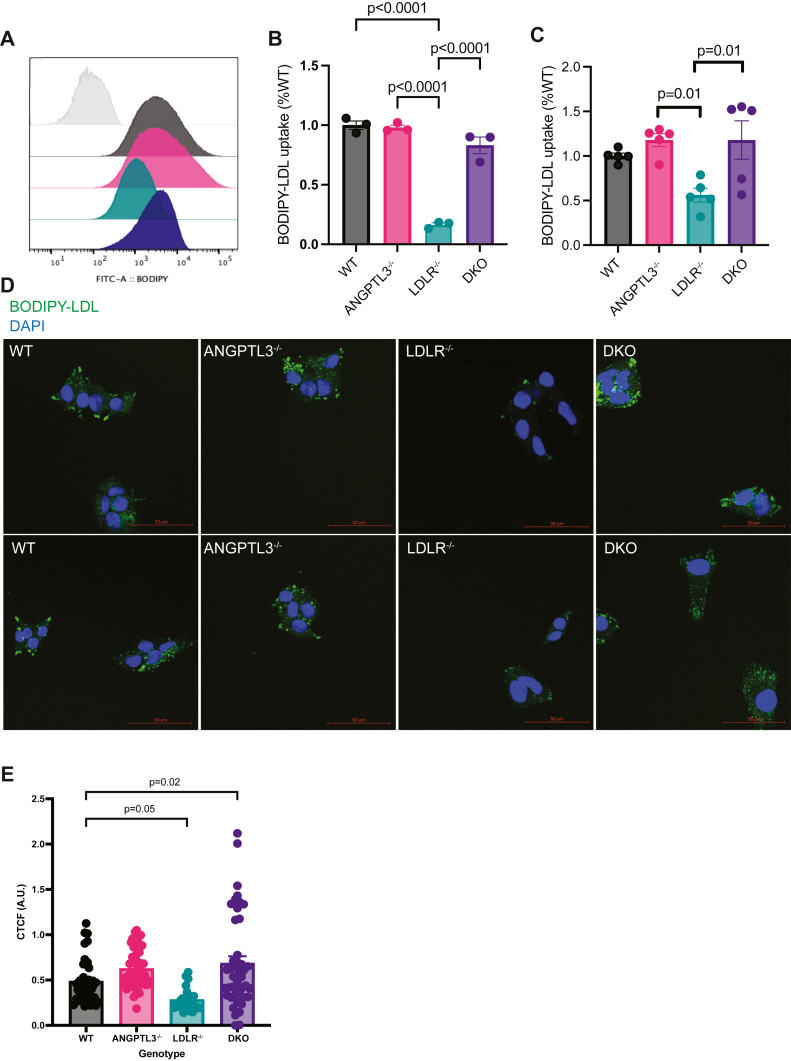
ANGPTL3 deficiency restores LDL uptake in the absence of LDLR. A: Representative FACS graph from cells treated with BODIPY-labeled LDL. Colors of distributions correspond to respective genotypes in (B) and (C). B, C: Cells were serum starved for 12 h prior to incubation with 10 μg/ml BODIPY-labeled LDL for 1 h (B) or 3 h (C). n = 3 or 5 wells per genotype in B and C, respectively. *P*-values as indicated by one-way ANOVA. D: Cells were treated as in (B), fixed, and stained with DAPI (blue) in addition to BODIPY-LDL treatment (green). Each column contains 2 representative images per genotype. The scalebar represents 50 μm. E: Quantification of corrected total cell fluorescence (CTCF) from experiment in (D). Images were collected from across n = 3 wells per genotype. DKO = *ANGPTL3*^*−/−*^*;LDLR*^*−/−*^ HepG2 cell line. *P*-values as indicated by one-way ANOVA. ANGPTL3, angiopoietin-like protein 3; FACS, fluorescence-activated cell sorting; LDLR, low-density lipoprotein receptor.

Given evidence of a late- and/or postsecretory mechanism of ApoB100 regulation in ANGPTL3-deficient cells lacking LDLR, we next wanted to determine whether these cells displayed any differences in hepatic uptake of circulating LDL. To address this question, we used flow cytometry (Fig. 7A–C) and confocal microscopy (Fig. 7D, E) to assess uptake of BODIPY-labeled LDL. As expected, *LDLR*^*−/−*^ cells exhibited reduced BODIPY-LDL uptake using both methods. Notably, this reduction was completely reversed in *ANGPTL3*^*−/−*^*;LDLR*^*−/−*^ cells, affirming prior observations of an LDLR-independent pathway for LDL clearance in the context of ANGPTL3 inhibition. In the setting of a post-Golgi regulatory site of ApoB100 degradation, these LDL uptake data suggest that enhanced hepatic clearance by an LDLR-independent pathway is important for LDL lowering when both ANGPTL3 and LDLR are absent.

## Discussion

**Fig. 8:**
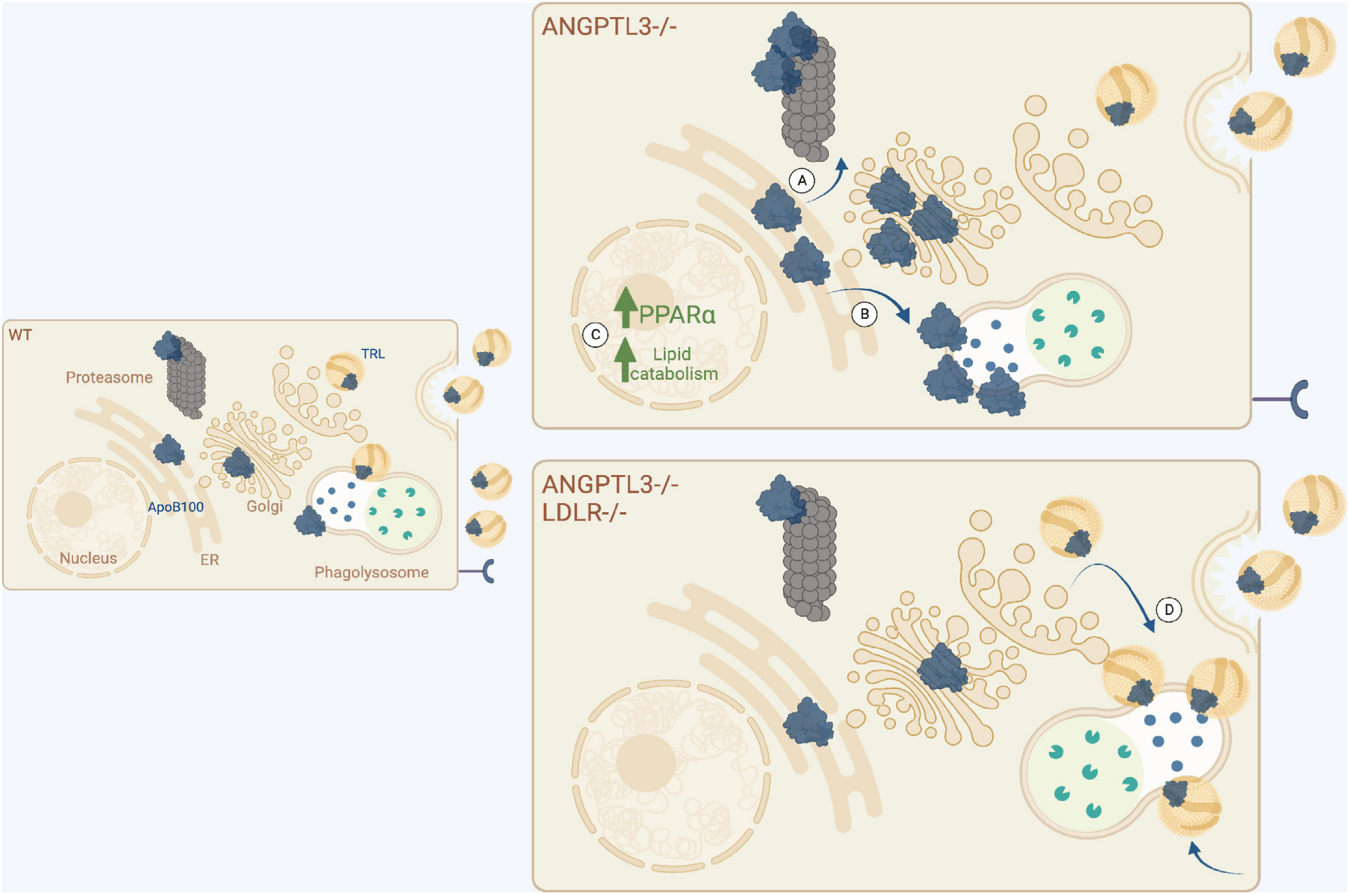
LDLR status determines how ApoB100 is degraded in ANGPTL3 deficiency. ANGPTL3-deficient HepG2 cells expressing LDLR increase both (A) chaperone-mediated proteasomal degradation and (B) lysosomal degradation of ApoB100 in a pre-Golgi compartment. This increased turnover of ApoB100 is associated with impaired particle secretion and failure to accumulate neutral lipids, accompanied by global transcriptional changes in lipid metabolism (C). ANGPTL3-deficient HepG2 cells lacking LDLR employ a different combination of mechanisms, likely lysosomal, to regulate ApoB100 degradation in a post-Golgi compartment (D). ANGPTL3, angiopoietin-like protein 3; ApoB100, apolipoprotein B100; ER, endoplasmic reticulum; LDLR, low-density lipoprotein receptor; TRL, triglyceride-rich lipoprotein particle.

Our findings provide several new insights. First, we demonstrate that intracellular ANGPTL3 deficiency modulates hepatic assembly and secretion of TG-enriched ApoB100-containing lipoproteins. Second, we describe associated adaptive changes in lipid metabolic homeostasis in ANGPTL3-deficient HepG2 cells. Third, and to the best of our knowledge, our findings provide the first evidence for two distinct pathways by which ANGPTL3 regulates ApoB100 synthesis and secretion in a manner dependent on LDLR functional status (Fig. 8). In the presence of LDLR, ANGPTL3-deficient cells employ a combination of mechanisms early in presecretory transit to increase ApoB100 degradation, consistent with observations of decreased particle secretion. In the absence of LDLR, ANGPTL3-deficient cells demonstrate increased ApoB100 degradation in a post-Golgi compartment. Furthermore, we demonstrate transcript-level evidence with functional validation supporting the notion that enhanced lipid catabolism prevents hepatic neutral lipid accumulation despite impaired particle assembly and secretion in *ANGPTL3*^*−/−*^ cells expressing LDLR.

Our radiolabeling experiments in *ANGPTL3*^*−/−*^ HepG2 cells revealed reduced secretion of newly synthesized ApoB100, consistent with previous cell culture experiments and also with studies of humans with lifelong genetic deficiency of ANGPTL3 (33, 34). With regards to LDL clearance, studies of humans (including those with familial hypercholesterolemia (FH)) receiving evinacumab have yielded similar results, demonstrating LDL lowering independently of LDLR (12, 30, 31, 35, 36). Reeskamp *et al.* (31) noted evinacumab lowered VLDL-ApoB secretion from baseline in two FH participants with LDLR null variants but not in two participants carrying LDLR defective variants. In contrast, mouse models employing pharmacologic inhibition of circulating ANGPTL3, demonstrated no effect on VLDL-ApoB production despite reductions in hepatic VLDL-TG production (37). Our findings agree with the conclusions from Reeskamp *et al.*, which note that the effects of ANGPTL3 inhibition on VLDL production likely differ based on LDLR status, metabolic state, and whether intrahepatic or circulating ANGPTL3 is inhibited. It should be noted that there are reports of effects of the LDLR regulating hepatic ApoB-lipoprotein secretion, and these effects may be relevant to ANGPTL3 as discussed further below. However, additional work is needed to more fully describe the mechanisms by which inhibition of ANGPTL3 reduces ApoB100 secretion in the presence and absence of LDLR.

Upon observing that ANGPTL3 deficiency did not lead to differences in the intracellular accumulation of a newly synthesized pool of ApoB across multiple time points, we wondered whether the decrease in secretion could be explained by increased degradation of ApoB100. As reviewed previously, three major pathways for ApoB100 degradation have been demonstrated in vitro (38). The first involves chaperone-mediated diversion of insufficiently lipidated ApoB100 from the ER to the proteasome, which in our model makes a minor contribution to the lipoprotein secretion defect in *ANGPTL3*^*−/−*^ cells expressing LDLR (38). The second pathway involves presecretory lysosomal degradation of ApoB100 (38). Although the ER has been identified as an initial regulatory site, this process also encompasses post-ER presecretory proteolysis (38, 39). As part of this presecretory regulation, intracellular LDLR can act on ApoB100 to directly target nascent TRL to the lysosome for degradation (39, 40). This model, studied in primary hepatocytes, could be consistent with our observation that impairment of the lysosome using bafilomycin reduces ApoB100 degradation by *ANGPTL3*^*−/−*^ cells to wild type levels when LDLR is present. Additionally, these *ANGPTL3*^*−/−*^ cells express higher levels of autophagy markers at the transcript level. Although the brefeldin A experiment in Blasiole *et al.* suggests LDLR directs ApoB100 to degradation in a post-ER compartment, ER localization of LDLR has also been shown in a similar primary hepatocyte model to drastically reduce ApoB100 secretion (39, 40). These latter data would be consistent with our observation of increased pre-Golgi degradation and concomitantly reduced ApoB100 secretion in *ANGPTL3*^*−/−*^ HepG2 cells expressing LDLR. Lastly, the third main pathway of ApoB100 degradation also uses lysosomal machinery, but instead to internalize newly secreted ApoB100 (38). Postsecretory lysosomal degradation may be contributing in this fashion to enhance turnover in the endocytic compartment of *ANGPTL3*^*−/−*^*;LDLR*^*−/−*^ cells, given their ability to clear wild type levels of fluorescently labeled LDL from cell culture media. Though our data support a post-Golgi site of enhanced ApoB100 degradation for *ANGPTL3*^*−/−*^*;LDLR*^*−/−*^ cells (in contrast to that of *ANGPTL3*^*−/−*^ cells expressing LDLR), further studies are required to identify this reuptake mechanism and whether it involves increased endolysosomal turnover of ApoB100. In short, our data support a model where *ANGPTL3*^*−/−*^ cells rely on intracellular LDLR to increase presecretory ApoB100 degradation, but in the absence of LDLR, a late secretory or postsecretory mechanism enhances ApoB100 degradation with contributions from an alternative particle clearance pathway.

Given the impaired assembly and secretion of ApoB100-containing lipoproteins in ANGPTL3-deficient HepG2 cells, we asked what metabolic adaptations, if any, existed as a result. The results of our targeted mass spectrometry-based lipidomics demonstrated that ANGPTL3-deficient HepG2 cells fail to accumulate neutral lipids. These findings differ from those of Xu *et al.* (33), though there are some key differences between the Huh7 cells used in their studies and the HepG2 cells used here. Although HepG2 cells degrade a larger proportion of ApoB100 at baseline compared to Huh7 cells, HepG2 cells also produce a shift toward lower-density particles with oleate treatment, a characteristic not observed in Huh7 cells. Thus, we speculate that the failure to accumulate neutral lipids in our oleate-treated HepG2 model could be attributed to more effective lipidation of ApoB100.

Our findings bear discussion in the context of recent clinical trials testing pharmacologic ANGPTL3 inhibition for the treatment of refractory hyperlipidemia. Vupanorsen, the ASO targeting ANGPTL3, demonstrated dose-dependent increases in hepatic fat accumulation (16) while RNAi against the same target did not demonstrate such increases (19). Additionally, evinacumab’s inhibition of circulating ANGPTL3 markedly reduced plasma lipids without reports of elevated liver function tests or hepatic fat accumulation in participants with FH (30). Combining our current findings with our recent liver MRI studies of ANGPTL3-deficient humans (18), we conclude that inhibition of intrahepatic ANGPTL3 alone is not expected to lead to hepatic fat accumulation. Indeed, studies have suggested biomarkers—including increased ketone body production by ANGPTL3-deficient human participants—may reflect increased hepatic fatty acid beta-oxidation, in agreement with our results (41). Likewise, our RNA sequencing results demonstrated increased expression of genes encoding enzymes involved in lipid metabolism, including several PPARα targets suggestive of an apparent shunting of fatty acyl CoAs to the oxidative pathway. However, additional studies are required to formally validate potential changes in chromatin accessibility and transcription factor binding to determine whether this effect is direct and mediated by PPARα. Nonetheless, our findings support a role for ANGPTL3 in the reorganization of lipid substrates to decrease lipogenesis and increase fatty acid catabolism.

We recognize some limitations to our conclusions, including the observation that hepatocellular carcinoma lines demonstrate developmental, metabolic, and immune aberrancies one would not expect to see in vivo (42). Furthermore, these hepatoma-derived cell lines tend to produce underlipidated particles more closely resembling LDL than VLDL. Additionally, our experiments do not address key questions related to the effects of ANGPTL3 deficiency on circulating lipoproteins, including the important contributions of endothelial lipase to LDLR-independent LDL lowering (43, 44, 45). Despite these limitations, our data point to an important intracellular role for ANGPTL3 in regulating the hepatic metabolism of ApoB100-containing triglyceride-enriched lipoproteins.

## Data availability

All data generated and analyzed in this manuscript are available from the corresponding author upon request. All data are provided in this manuscript and supplementary information. RNA sequencing data have been deposited under accession GSE232045 and lipidomic data are included in the supplemental materials.

## Supplemental data

This article contains supplemental data.

## Conflict of interest

N. O. S. has received investigator-initiated research funding from Regeneron Pharmaceuticals related to ANGPTL3. I. J. G. has received consulting fees from Arrowhead Pharmaceuticals. All other authors have no relevant conflicts with the contents of this article.
