# Supplementary material for: ANGPTL3 deficiency impairs lipoprotein production and produces adaptive changes in hepatic lipid metabolism
Source: J Lipid Res. 2024 Jan 14;65(2):100500. doi: 10.1016/j.jlr.2024.100500 (PMC10875267; doi:10.1016/j.jlr.2024.100500)
Supplement: Supplemental Figures S1–S8 [file mmc1.pdf]

SUPPLEMENTAL MATERIAL

ANGPTL3 deficiency impairs lipoprotein production and produces adaptive changes in hepatic lipid metabolism

Kendall H. Burks, Yan Xie, Michael Gildea, In-Hyuk Jung, Sandip Mukherjee, Paul Lee, Upasana Pudupakkam, Ryan Wagoner, Ved Patel, Katherine Santana, Arturo Alisio, Ira J. Goldberg, Brian N. Finck, Edward A. Fisher, Nicholas O. Davidson, Nathan O. Stitziel

This file includes:

Figure S1. Generation of ANGPTL3 knockout HepG2 cell line

Figure S2. ANGPTL3 deficiency does not alter ApoB transcription in HepG2 cells

Figure S3. ANGPTL3 deficiency reduces ApoB secretion in a second knockout HepG2 clone

Figure S4. ANGPTL3 knockout HepG2 cells show decreased ApoB100 secretion upon treatment with high concentration of oleate

Figure S5. Validation of decreased TG secretion at additional time point and in second ANGPTL3 knockout HepG2 clone

Figure S6. Approximation of secreted particle size

Figure S7. RNAseq QC plots

Figure S8. Generation of LDLR knockout HepG2 line with and without ANGPTL3 knockout

A

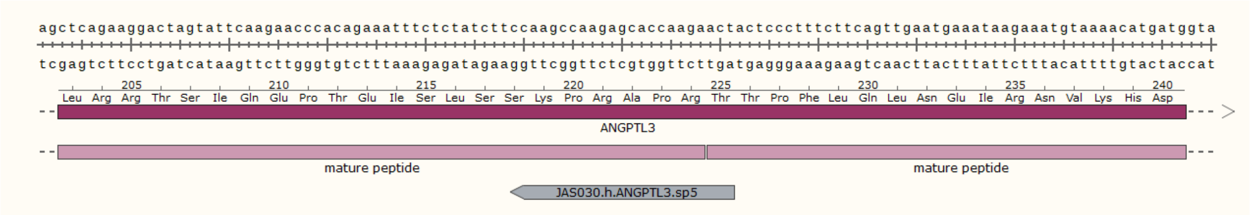

B

	JAS030 5A12	JAS030 5H7	Reference(Expasy)
**AMELX**	XY	XY	XY
**PentaE**	15, 20	15, 20	15, 20
**D21S11**	31, 29	29, 31	29, 31
**D19S433**	17.2 [15.2]	17.2 [15.2]	15.2
**D13S317**	9, 13	9, 13	9, 13
**TPOX**	9, 8	9, 8	8, 9
**D7S820**	10	10	10
**D5S818**	11	11	11, 12
**CSF1PO**	10, 11	10, 11	10, 11
**D16S539**	12, 13	12, 13	12, 13
**D3S1358**	15, 16	15, 16	15, 16
**TH01**	9	9	9
**D8S1179**	15, 16	15, 16	15, 16
**VWA**	17	17	17
**FGA**	22, 25	22, 25	22, 25
**D2S1338**	20, 19	20, 19	20, 19
**D18S51_#1**	13, 14	13, 14	13, 14
**PentaD**	13, 9	13, 9	13, 9

C

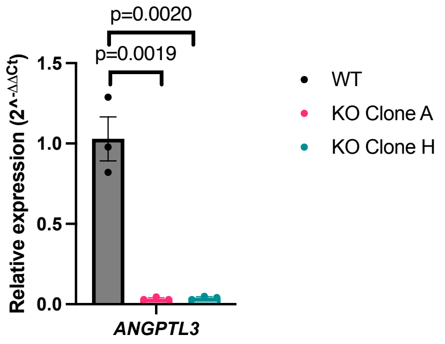

Figure S1. Generation of ANGPTL3 knockout HepG2 cell line. (**A**) Region of *ANGPTL3* targeted by CRISPR guide RNA. (**B**) STR profiling of *ANGPTL3*$^{-/-}$ clones. (**C**) qPCR validation of knockout at transcript level.

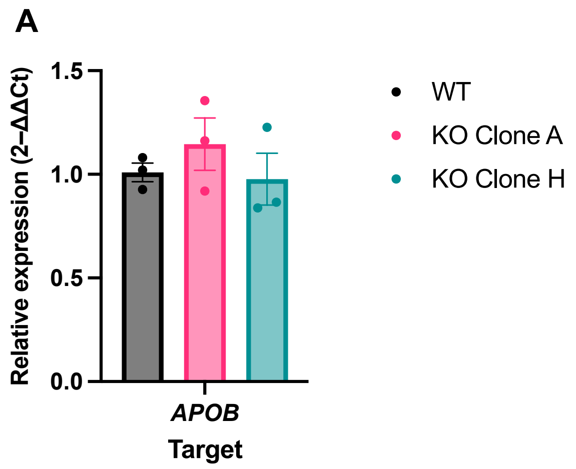

Figure S2. ANGPTL3 deficiency does not alter ApoB transcription in HepG2 cells. (A) qPCR showing no change in *APOB* mRNA when cells are treated with 600 μM oleic acid for up to 24 hr.

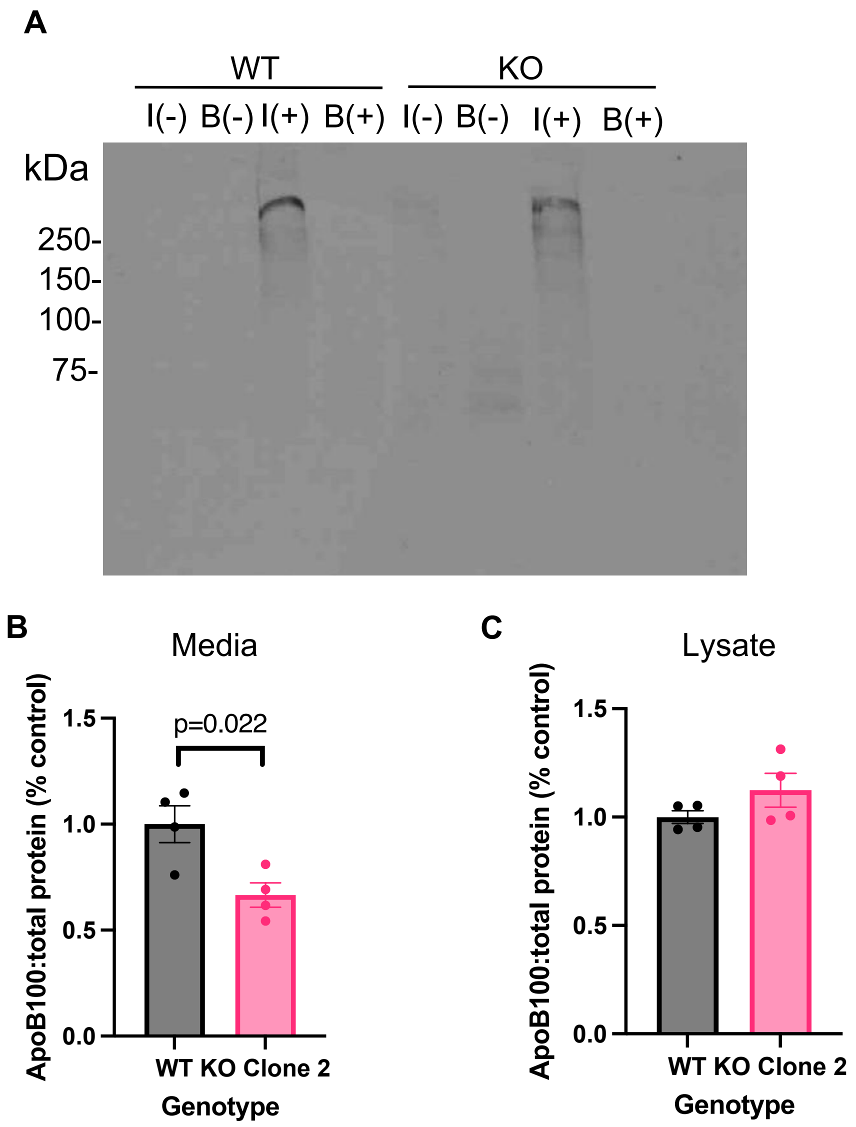

Figure S3. ANGPTL3 deficiency reduces ApoB secretion in a second knockout HepG2 clone. (A) ^{35}S radiolabeling experiment controls. ApoB100 pulldown from cell media performed as in Figure 1. I = isotype control for ApoB antibody pulldown. B = pulldown with ApoB antibody on an independent knockout clone. ^{35}S radiolabeling experiment with ApoB100 pulldown from cell culture media (**B**) or cell lysates (**C**) performed as in Figure 1 on an independent knockout clone.

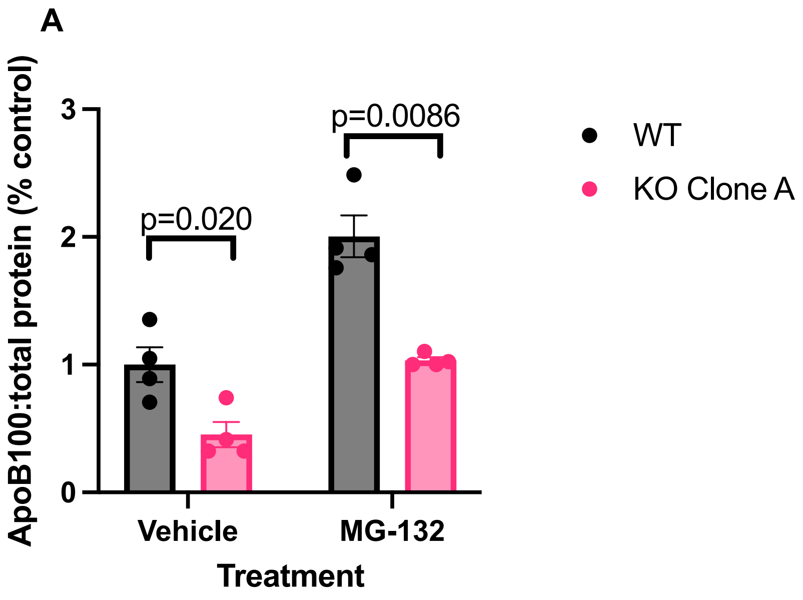

Figure S4. ANGPTL3 knockout HepG2 cells show decreased ApoB100 secretion upon treatment with high concentration of oleate. (A) ^{35}S radiolabeling experiment with ApoB100 pulldown from cell media performed as in Figure 1 with 600 μM oleate.

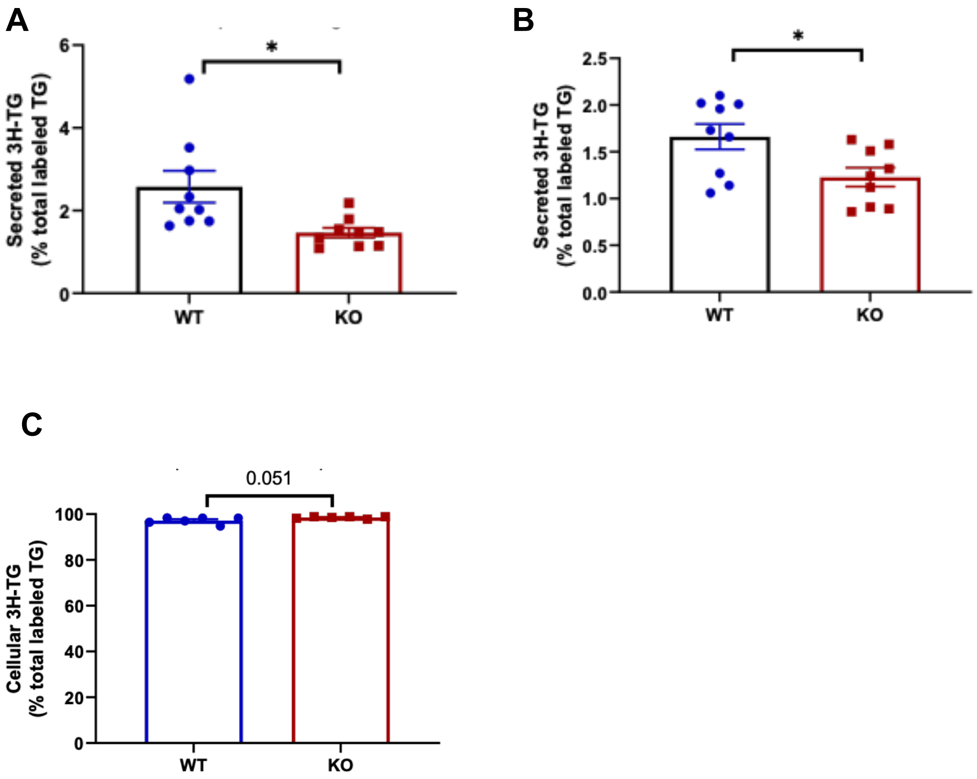

Figure S5. Validation of decreased TG secretion at additional time point and in second ANGPTL3 knockout HepG2 clone. (A) Experiment performed as in Figure 3. Medium was collected at 0 and 16 hours. **(B)** Experiment performed as in Figure 3 using second *ANGPTL3*$^{-/-}$ HepG2 clone. Medium was collected at 0 and 4 hours. (**C**) Experiment performed as in Figure 3. TGs were extracted from cell lysates at the end of a 4 hour pulse period and counted.

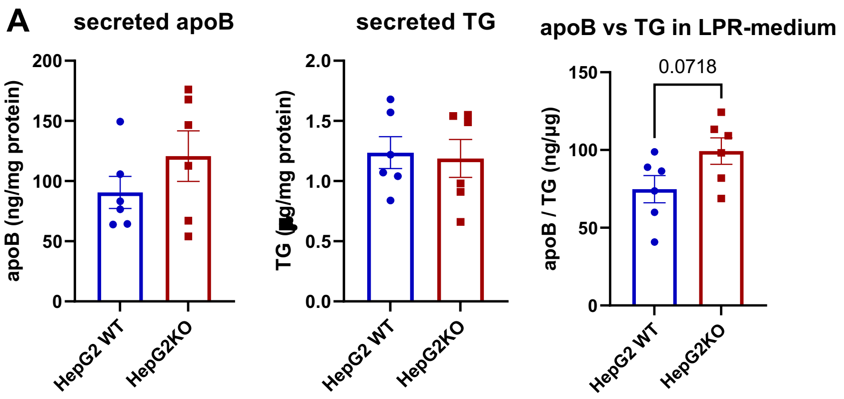

Figure S6. Approximation of secreted particle size. (A) Cells were cultured overnight in media containing 200 µM oleic acid-BSA. Lipoproteins were floated to a density of <1.21g/mL. ApoB100 mass was determined by ELISA and corrected to cellular protein content. TG mass was determined following Folch extraction using colorimetric assay. The ratio between apoB and TG was used to approximate particle size.

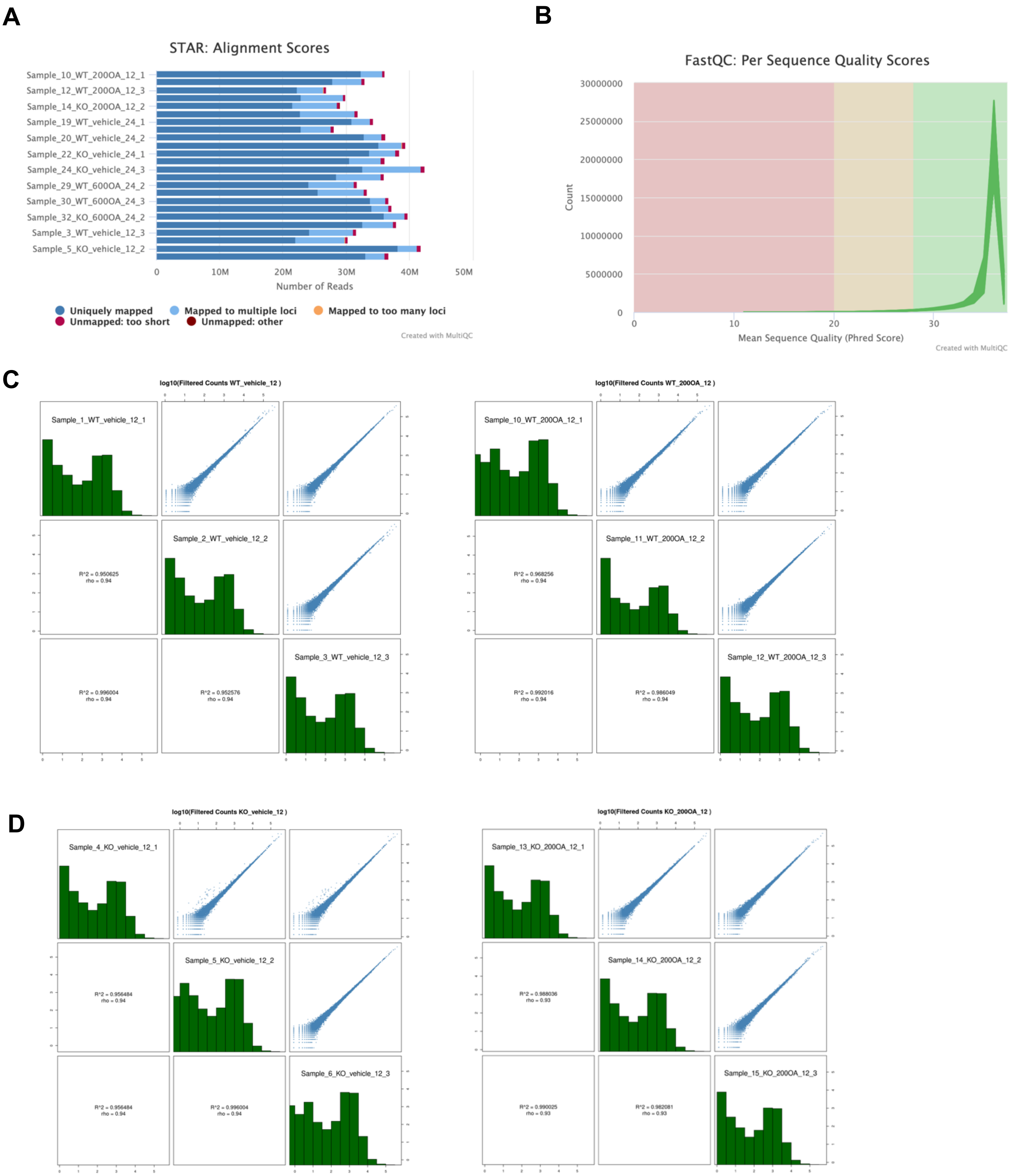

Fig S7. RNAseq QC plots. (A) STAR alignment score showing the number of reads uniquely mapped, mapped to multiple loci, or unmapped. **(B)** FastQC per sequence quality scores showing the number of reads with average quality scores. **(C)** WT replicate scatterplots. **(D)** *ANGPTL3*$^{-/-}$ replicate scatterplots.

A

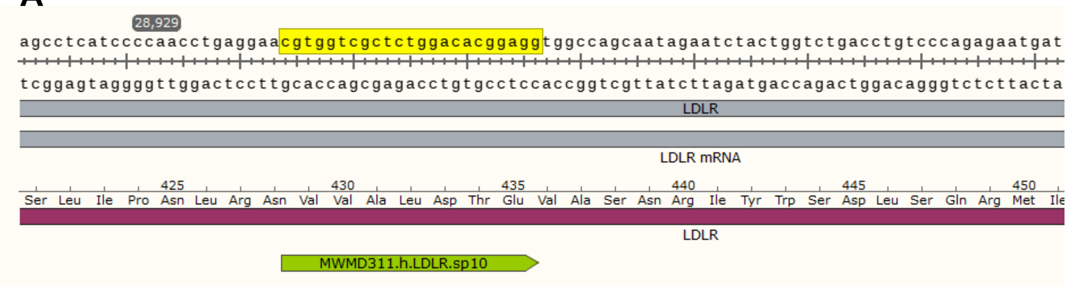

B

STR	A1	A3	A9	D6	D7	E4	ATCC HepG2
D19S433	17.2	17.2	17.2	17.2	17.2	17.2	17.2
D2S1338	20, 19	20, 19	20, 19	20, 19	20, 19	20, 19	20, 19
FGA	22, 25	22, 25	22, 25	25, 22	22, 25	22, 25	22, 25
PentaE	15, 20	15, 20	15, 20	15, 20	15, 20	15, 20	15, 20
CSF1PO	11, 10	11, 10	10, 11	11, 10	11, 10	10, 11	10, 11
D13S317	13, 9	9, 13	9, 13	13, 9	9, 13	9, 13	9, 13
D3S1358	15, 16	15, 16	16, 15	16, 15	15, 16	15, 16	15, 16
D8S1179	15, 16	15, 16	15, 16	16, 15	15, 16	15, 16	15, 16
TH01	9	9	9	9	9	9	9
AMELX	2, 1	1, 2	1, 2	1,2	1, 2	1, 2	1, 2
D16S539	12, 13	12, 13	12, 13	12, 13	12, 13	12, 13	12, 13
D21S11	29, 31	29, 31	31, 29	31, 29	31, 29	29, 30	29, 31
D5S818	12, 11	11, 12	11, 12	11, 12	11, 12	11, 12	11, 12
D7S820	10	10	10	10	10	10	10
TPOX	9, 8	9, 8	9, 8	9, 8	9, 8	9, 8	9, 8

C

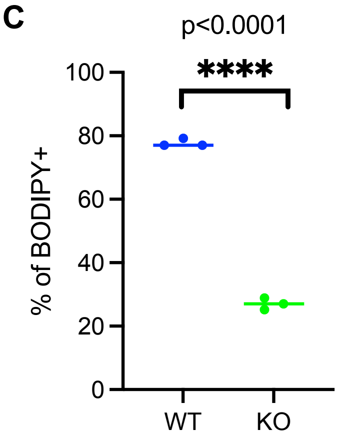

D

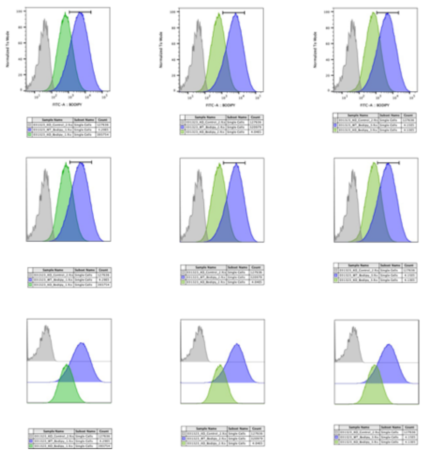

Figure S8. Generation of LDLR knockout HepG2 line with and without ANGPTL3 knockout. (**A**) Region of *LDLR* targeted by CRISPR guide RNA. (**B**) STR profiling of *LDLR*$^{-/-}$ and *ANGPTL3*$^{-/-}$;*LDLR*$^{-/-}$ clones. (**C**) Functional validation of LDLR knockout by BODIPY-LDL uptake, performed as in Figure 7. (**D**) Representative FACS plots from BODPY-LDL uptake experiment.
